# From diagnosis to survivorship addressing the sexuality of women during cancer

**DOI:** 10.1093/oncolo/oyae242

**Published:** 2024-09-12

**Authors:** Rebekah Kaufman, Laila Agrawal, Eleonora Teplinsky, Lauren Kiel, Oyepeju Abioye, Narjust Florez

**Affiliations:** Department of Thoracic Oncology, Dana Farber Cancer Institute, Boston, MA 02215, United States; Medical Oncology, Norton Cancer Institute, Louisville, KY 40241, United States; Medical Oncology, Valley Mount Sinai Comprehensive Cancer Center, Paramus, NJ 07652, United States; Department of Thoracic Oncology, Dana Farber Cancer Institute, Boston, MA 02215, United States; Department of Thoracic Oncology, Dana Farber Cancer Institute, Boston, MA 02215, United States; Department of Thoracic Oncology, Dana Farber Cancer Institute, Boston, MA 02215, United States

**Keywords:** sexuality, social media, oncology

## Abstract

For women diagnosed with cancer, side effects affecting their sexuality are extremely common and can be distressing and life-changing; however, most women are left in the dark without any guidance from their oncology teams regarding possible side effects and treatment options. American Society of Clinical Oncology clinical guidelines provide guidance on the recommended assessments related to the domains of sexual function and their respective interventions. Despite the existence of these guidelines, the reality is that only a few women with cancer are asked about sexual concerns that result from cancer treatments. Common barriers to sexuality discussion reported by oncology providers include a lack of qualification and knowledge, not having a place to refer patients, and not knowing how to start the conversation. Social media remains a widely untapped resource regarding sexuality and cancer interventions, as people are increasingly turning to social media for health information and advice. This may be especially relevant for sexuality, as oncologists may not feel comfortable or well-trained to discuss the topic, and patients may be reluctant to bring up sexual concerns during their visits. Social media can play a critical role in studying sexual health and in sexuality interventions, particularly in adolescent and young adult patients with cancer. Here, we discuss the lack of inclusion regarding sexuality in oncology, the rates of sexual dysfunction in patients with cancer, treatment options for common sexual concerns, how to utilize the reach of various social media channels, and provide patient and provider resources.

## Teaser

Studies show that many women with cancer experience sexual concerns. The goal of this review is to bring awareness to the rates of sexual dysfunction in women with cancer, highlight treatment guidelines for common sexual health side effects, and provide resources for patients and providers.

**Implications for Practice**

Sexuality is often an avoided topic by oncology providers. Lack of knowledge, resources, and training are commonly reported reasons for avoiding sexuality discussions. However, studies report that many women with cancer experience sexual concerns and that sexual health is acutely related to quality of life. Given the importance of sexuality to overall well-being, we hope this review will bring awareness to the rates of sexual dysfunction in women with cancer, highlight treatment guidelines for common sexual health side effects, and provide resources for patients and providers.

## Sexual health and cancer: the dismissed concern

For women diagnosed with cancer, sexual dysfunction is associated with higher symptom burden, anxiety, and depression. However, most women are left in the dark without any guidance from their oncology teams regarding potential side effects and treatment options.^1-3^ As a result, sexual dysfunction often goes untreated, impacting quality of life, mental and physical health, and relationships.^1,3^

Sexual dysfunction in women after cancer treatment has been well documented. Persistent sexual concerns, as a result of oncologic treatment and psychological distress, have been reported in up to 90% of women with gynecological cancer, up to 75% of women diagnosed with breast cancer, and 77% of women with lung cancer.^1,4-7^ Certain cancer treatments can result in specific sexual health toxicities. For example, 29%-49% of female stem cell transplant recipients may experience gynecological graft versus host disease symptoms, such as vaginal dryness, dyspareunia, vulvovaginal scarring, and vaginal stenosis.^8^

Multiple professional medical organizations have recognized the importance of sexuality and wellness after cancer diagnosis and have issued guidelines or consensus statements regarding the assessment and treatment of sexual dysfunction in patients with cancer.^9-11^ These guidelines recommend regular assessments and treatments of sexuality throughout cancer diagnosis, treatment, and follow-up.

Despite the existence of these guidelines, the reality is that only a few women with cancer are asked about sexuality concerns that result from cancer treatments.^3^ A survey of patients in a radiation oncology clinic reported that 87% had the impact on sexual function, however only 27.9% were ever asked about sexuality by a medical professional.^12^ Gender disparities exist within this topic, in that 53% of men vs 22% of women with cancer were directly assessed for sexual concerns, fueling the already existing gender disparities in cancer care.^12^ Research from Living Beyond Breast Cancer reveals that patients feel oncology teams do not address sexual health and 64% of young women with breast cancer reported sexuality concerns that their provider was unable to address.^13^

Oncology professionals can meet this need by addressing sexuality concerns throughout the spectrum of cancer care, offering mitigating strategies and referrals to specialists when needed. Cancer centers can work to address this unmet need by developing sexual health programs where patients can receive a bio-psycho-social assessment and treatment options.

This review will address the barriers medical professionals face in addressing sexuality in women, the incidence of sexual dysfunction in women with cancer, treatment options, and the potential role of social media in mitigating these issues.

## Lack of inclusion in routine oncology care

**Figure 1.**
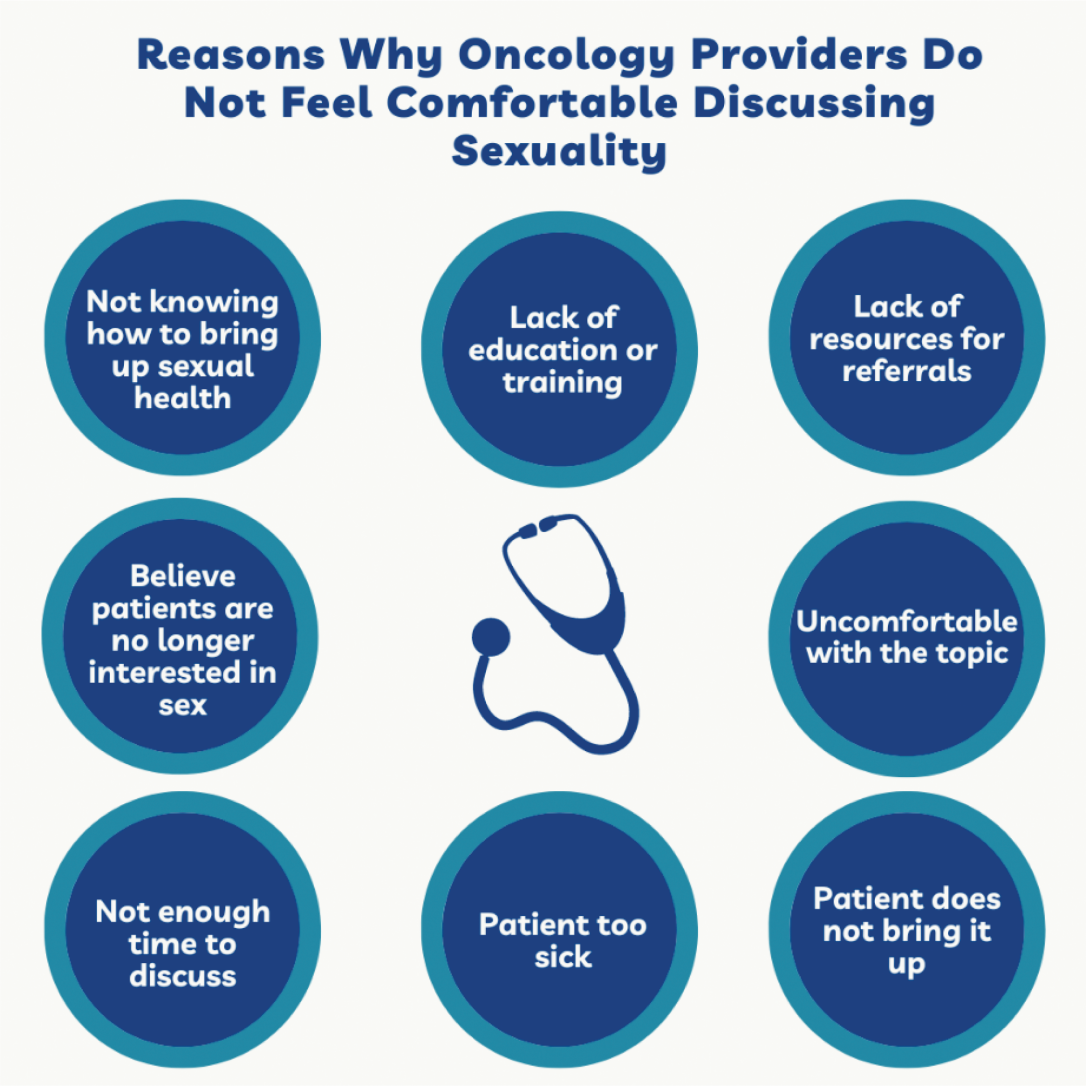
Top reasons why oncology providers do not feel comfortable discussing sexuality.

Despite the importance of sexuality to overall well-being, sexuality in cancer care is often a forgotten and avoided subject.^2,14^ Research reports that sexuality concerns are avoided topics by providers.^2^ Providers cite barriers such as not feeling qualified or prepared to discuss sexuality with oncology patients, lack of time, patient resources for referrals, lack of knowledge, and not knowing how to bring up the subject as reasons for why they do not discuss sexuality (Figure 1).^2,15,16^ In addition, intersectional identities impact the discussion of sexuality during routine oncology care. Patients who are women, LGBTQ+, and patients who are religious minorities or religiously observant are less likely to receive discussions about sexuality as bias, lack of knowledge and training, or concern about offending patients in these groups can be greater.^14,16-19^

### Gender Bias

Studies indicate that disparities exist regarding the discussion of sexuality. In France, a nationwide survey found a statistically significant difference (*P* < .001) in the discussion of sexuality for patients with cancer, reporting that 11.1% of women and 36.7% of men with cancer had sexual health discussions with a provider.^17^ The likelihood of discussion was also impacted by cancer type.^17^ Patients with prostate and cervical cancers had a higher chance of receiving discussions, though the likelihood was higher in patients with prostate cancer (56.3%) compared to patients with cervical cancer (39.6%).^17^ These findings were reaffirmed by data from the US reporting that sexuality was assessed at consult for 13% of patients with cervical cancer and 89% of patients with prostate cancer.^20^ More so, other studies reveal that discussions varied by cancer type: 80% of patients with breast cancer and 82% of patients with blood or marrow cancer reported having no discussion of sexuality at all.^18^ Notably, conversations about the sexuality impact of treatments were more likely to be brought up by female oncologists (*P* = .02).^18^

### LGBTQ+ patients

Sexual orientation and gender identity data are not routinely collected in oncology care, leading to a general lack of information on patients who identify as sexual and gender minorities (SGM).^14^ Providers may feel uncomfortable or untrained in addressing sexuality in patients who identify as SGM, perpetuating the marginalization that SGM groups continue to experience.^14^ More so, lack of provider knowledge and bias is often exacerbated in transgender/gender diverse (TGD) individuals because of insufficient research centering patients who are TGD and greater implicit biases from providers.^14^ In a qualitative study on the reasons why providers did not discuss sexuality with SGM patients, not knowing how to bring up the topic, insufficient time in appointments, bias against LGBTQ+ individuals, not having the tools to help, and fear of offending the patient were cited as reasons by providers.^19^ For TGD individuals, lack of provider knowledge and formalized clinical guidelines significantly impacts gender-affirming care practices during cancer treatment, adding to the isolation that many of these patients experience.^21^ Gender-affirming care is a part of holistic sexual and reproductive health practices, indicating the importance of provider education and subsequent discussion of these topics in oncology care.^22^

### Religious minorities or religiously observant

Religion and spirituality can have a positive impact on cancer care and are important to many patients.^23^ Researchers have emphasized the importance of culture when discussing sexuality.^24^ In a study of 433 medical oncologists, 23.7% reported a patient’s culture or religion as a reason for avoiding a sexual discussion.^16^ As religions have different views on sexuality and sexual health, discussions and treatment should be individualized to the patient’s needs.^25^ Cultural humility is a core tenant of equitable care, and as such, learning about a patient’s religious practice is critical to understanding the psychosocial and sexual health needs of patients.^26^

## Status of research about sexual health and cancer

### Status of research

Despite overall low reports of discussions for women,^17^ data on the incidence, types of sexual dysfunction, and rates of where it is addressed highlight the importance of improving sexuality care for patients with cancer. In a meta-analysis encompassing 5483 women with cancer, the prevalence of sexual dysfunction, encompassing concerns with arousal, orgasm, interest, and pain, was 66% based on the female sexual dysfunction index (FSFI).^27^ Notably, there are inconsistencies across reports; in a systematic review, there was a significant variation in the reported incidence of sexual dysfunction, ranging from 30% to 80%.^28^ Further, it is reported that the risk of developing sexual dysfunction was 2.7 to 3.5 times higher in women with cancer than those who did not have cancer.^28^

Women with cancer in reproductive organs often report the highest level of sexual dysfunction^27,28^; studies estimate that 90% of patients with gynecologic cancer experience changes in sexual experiences, preferences, and are at increased risk of dyspareunia and vaginal dryness.^29,30^ In cancer types where sexual health is less likely to be discussed, such as lung, breast, colorectal, and head and neck cancer, the prevalence and areas of sexuality concerns have also been reported.^6,7,31^ In the SHAWL study, 77% of women with lung cancer reported sexual dysfunction.^6^ Among patients affected by breast cancer it is reported that that 75% of women report sexual concerns^7^ and 89.5% report changes to sexuality from treatment.^32^ Sexual function has been reported as the most significantly affected quality-of-life measure during breast cancer treatment, with the most common sexual concerns including pain with penetration, vulvovaginal dryness, and decreased desire and arousal.^33,34^ Research centering on patients with colorectal and anal cancer revealed 98% of patients had FSFI scores associated with significant sexual dysfunction.^31^ Sexuality is also impacted in head and neck cancer as treatment can involve surgery, chemotherapy, and radiation, which can be disfiguring, impact saliva production, and induce hormonal changes; in fact, sexuality and body image are altered in 79.2% of female head and neck cancer survivors.^35^

## Treatment of sexual health

The American Society of Clinical Oncology (ASCO) guidelines recommend that medical professionals should assess the following domains of sexual function and recommended interventions: the genitourinary syndrome of menopause, sexual response—libido, arousal, and orgasm, dyspareunia, and psychosocial concerns including depression, anxiety, body image concerns, and relationship issues (Table 1).^9^

**Table 1.** Treatment recommendations.

Domain	Professional society recommendations
Assessment	ASCO: Discussion initiated by healthcare team regarding sexual health and dysfunction resulting from cancer or its treatment at time of diagnosis and continue to readdress during treatment course and survivorship NCCN: Ask about sexual health at regular intervals
Libido	ASCO: Psychosocial or psychosexual counseling, couple’s interventions, regular stimulation (including masturbation), flibanserin for pre-menopausal women NCCN: Psychosocial counseling, Discussion of medications including androgens, bupropion, buspirone, flibanserin, and bremelanotide
Genitourinary syndrome of menopause	ASCO: Vaginal moisturizers, lubricants, and vaginal estrogen NCCN: Non-hormonal treatments (vaginal moisturizers, gels, hyaluronic acid, oils), lubricants for sexual activity, local estrogen, and DHEA NAMS/ISSWSH: Non-hormonal therapies are generally first-line therapy ACOG: Silicone, polycarbophil, and water-based lubricants such as hyaluronic acid, polyacrylic acid, and vitamin E and D suppositories should be considered first-line treatment for urogenital symptoms in individuals with a history of estrogen-dependent breast cancer
Dyspareunia	ASCO: Cognitive-behavioral therapy, pelvic floor therapy, exercise, vaginal dilators NCCN: Topical vaginal therapies, vaginal dilators, ospemifene, DHEA, pelvic physical therapy, topical analgesics
Orgasm (less intense, difficulty achieving, pain)	NCCN: Discuss options including vibrator or clitoral stimulatory device with referral to appropriate specialist and consider pelvic floor physical therapy
Vaginal hormones in hormone sensitive breast cancer	ASCO: For those who do not respond or whose symptoms are more severe at presentation, *low-dose* vaginal estrogen can be used. For women with hormone-positive breast cancer who are symptomatic and not responding to conservative measures, *low-dose* vaginal estrogen can be considered after a *thorough* discussion *of risks and benefits.* NCCN: Limited data in breast cancer survivors suggest minimal systemic absorption with rings and suppositories. Therefore, if estrogen-based treatment is warranted, rings and suppositories are preferred over creams for survivors of hormonally sensitive tumors. NAMS/ISSWSH: Women with severe symptoms where nonhormone treatments have failed may still be candidates for local hormone therapies after review with the woman’s oncologist vs consider switching to tamoxifen ACOG: If non-hormonal treatments have failed to adequately address symptoms, after discussion of risks and benefits, low-dose vaginal estrogen may be used in individuals with a history of breast cancer, including those taking tamoxifen. For individuals taking aromatase inhibitors (Ais), low-dose vaginal estrogen can be used after shared decision-making between the patient, gynecologist, and oncologist.
	ASCO: American Society of Clinical Oncology NCCN: National Comprehensive Center Network NAMS/ISSWSH: North American Menopause Society/International Society for the Study of Women’s Sexual Health ACOG: American College of Obstetrics and Gynecology

### Assessment

Sexual function and distress related to sexual concerns should be assessed in female patients with cancer regularly. There are multiple methods available to assess sexuality, including asking during routine visits, utilizing a paper checklist, and incorporating it into standard side effect assessments.^36^ One helpful tool is to use a ubiquity statement to normalize the discussion of sexuality concerns, such as “many women on aromatase inhibitors experience vaginal dryness, low libido, or changes in sexual health followed with “have you experienced these concerns?.”^13^ It is important that this is a regular discussion over the course of follow-up.

### Libido

Low libido is a common concern after cancer treatment. If this is distressing to the patient, then interventions can be recommended. Psychosocial counseling, sex therapy, and couples counseling are first-line treatments for low libido.^9^ Sensate focus, mindfulness, exercise, and hypnosis have also shown benefit.^37-40^ A recent systematic review of randomized trials identified that multi-modal interventions, including education, acceptance, mindfulness, and communication/relationship skills, effectively improved sexual function.^39^ Outside of these modalities, flibanserin (a dual serotonin 1A receptor agonist/2A receptor antagonist) may improve libido, the number satisfying sexual events, and decrease distress in women with breast cancer on endocrine therapy.^41^ Bupropion has been suggested as another agent to help with low libido; however, a randomized trial of the anti-depressant bupropion compared to placebo in patients with breast cancer showed no difference in desire score on the FSFI.^42^ The lack of difference may have been due to untreated physical concerns, such as vaginal dryness, decreased lubrication, and pain with penetration.^42^ This underscores the importance of the bio-psycho-social assessment and treatment of sexual dysfunction in women with cancer in a holistic manner.

### Genitourinary syndrome of menopause

The genitourinary syndrome of menopause (GSM) includes vaginal and vulvar dryness, decreased lubrication, vaginal narrowing or shortening, and urinary symptoms.^11,43-45^ GSM is common in patients who are affected by cancer and is caused by cancer treatments and menopause.^44^ Non-hormonal moisturizers, lubricants, and low-dose local vaginal hormones are used to treat GSM. Use of a vaginal moisturizer containing hyaluronic acid three to five times per week can improve vulvovaginal tissue and sexual function.^46,47^ Patients should be educated about the appropriate use of vaginal lubricants, including recommendations of products with the appropriate pH of around 4.5 for vaginal products and osmolality of <1200 mOsm/kg as recommended by the World Health Organization, as well as the differences between water and silicone-based lubricants.^48^ The use of local vaginal hormones in hormone receptor-positive breast cancer is included in all the noted guidelines after a discussion of risks and benefits with the patient. Both a cohort study and a nested case-control study did not show an increased risk of cancer recurrence in patients with breast cancer on endocrine therapy who used vaginal estrogen when comparing quantity of use to non-use respectively.^49,50^ The Danish observational cohort study reported that while, overall, women with breast cancer who used local estrogen did not have an increased risk of cancer recurrence, the subgroup of patients on aromatase inhibitors did have an increased risk of cancer recurrence (1.39 [95% CI = 1.04-1.85), without an increase in mortality.^51^ Limitations of this study include that the doses of vaginal estrogen were not reported and may have been higher than the currently used low-dose vaginal estrogen, the study period pre-dated HER2 testing, which significantly affects the risk of recurrence, and that many patients were treated without any endocrine therapy.^51^ Another more recent retrospective study evaluated a group of 42 113 women diagnosed with genitourinary syndrome of menopause (GSM) following a breast cancer diagnosis, of which 3.9% utilized prescriptions for vaginal estrogen.^52^ The risk of recurrence was comparable between vaginal estrogen and the control group. Among the 10 584 patients with documented estrogen receptor-positive breast cancer, 3.9% used vaginal estrogen, and the risk of recurrence was similar. In a very small subgroup of patients identified to have concurrent prescriptions for vaginal estrogen and an aromatase inhibitor, there was an increased risk of breast cancer recurrence; however, results were not controlled for stage, grade, or nodal status.^52^ Lastly, a recent study of 49 237 women with breast cancer with 5% of patients using vaginal estrogen therapy found no increased risk of breast cancer-specific mortality (HR, 0.77; 95% CI, 0.63-0.94).^53^

### Dyspareunia

Pain with penetration or dyspareunia has been reported in patients with breast cancer, gynecologic cancers, lung cancer, and colorectal cancer^6,30,31,54,55^ Dyspareunia can be caused by vaginal stenosis or narrowing, insertional dyspareunia, or pelvic floor dysfunction.^56,57^ Vaginal dilators help treat vaginal stenosis and should be recommended after pelvic radiation to reduce the risk of vaginal stenosis.^56^ Pelvic floor dysfunction frequently contributes to dyspareunia and can be treated with pelvic floor physical therapy and, in some cases surgery.^57^ For patients with insertional dyspareunia, the application of 4% topical aqueous lidocaine can reduce symptoms of pain at the time of penetration, however, lidocaine may cause reduced sensitivity for a penetrating partner.^58-60^

### Sexual positions that limit discomfort

After a cancer diagnosis, certain sex positions that were once pleasurable may now cause symptoms such as fatigue, pain and/or discomfort, and shortness of breath.^61^ People experiencing decreased energy from diagnosis and treatment may benefit from side-lying positions and/or having their partner assume the active role.^61^ This may be particularly beneficial for patients with breast cancer or individuals with ostomy bags to avoid rubbing against sensitive areas.^61^ More so, patients with breathing concerns may want to avoid positions where they are lying flat on their back and may benefit from side-by-side and superior positions, which allow for better breathing and control.^61,62^ It is important that partners communicate with each other and try out different ways and positions to create an enjoyable experience. As symptoms may change over time, there is not one position that will be right for everyone every time. Pillows of different shapes and forms are a helpful tool to provide additional support to painful or uncomfortable areas.^63^ Certain areas that were once erogenous zones, such as the breasts, may no longer be after cancer, and identifying other pleasurable areas in the body should be encouraged by providers as patients learn new areas of stimulation and discover their new body during or after cancer treatments.^63^

### Complementary medicine

Sexual concerns often occur due to changes in both physical and mental health.^5,64^ A cancer diagnosis can result in feelings and symptoms such as anxiety, depression, fear, guilt, anger, and loneliness, which can subsequently impact body image, sexual desire, and sexual functioning.^64^ Medications often used to treat anxiety and depression can result in sexual side effects such as decreased libido or inhibition of orgasm. This is further exacerbated in patients with cancer who identify as LGBTQI+ as they experience worse mental health outcomes, higher levels of depression and anxiety, discrimination, and cancer-related distress.^65^

The ASCO Clinical Practice guideline recommends that psychosocial and/or psychosexual counseling be offered to all patients with cancer, aiming to improve overall sexual functioning, body image perception, intimacy, and relationship issues.^9^ Studies utilizing cognitive-behavioral therapy, couples-based sexual education, self-healing training, and group therapy with guided imagery have shown benefit in female survivors of cancer.^66^ Pelvic floor physical therapy may be helpful for patients experiencing symptoms of potential pelvic floor dysfunction such as persistent pain and urinary and/or fecal leakage.^66^ Integrative medicine options such as acupuncture, slow-breathing techniques and hypnosis have also shown some benefit and are overall low-cost interventions.^67-69^ Throughout such ventures, though, lack of insurance coverage and availability of trained and knowledgeable professionals remains a barrier to implementation.^66^

## Sexuality and social media

Disseminating existing treatment guidelines for sexual health in a way that is accessible and understandable to patients is vital.^9^ Notably, people are increasingly turning to social media for health information and advice.^70,71^ More so, it is theorized that traditional approaches to cancer care will not be able to manage the increase in survivorship care demands,^72^ especially given the rising number of cancer diagnoses each year and the increasing incidence of cancer in younger adults.^73^ This may be especially relevant to sexuality, as oncologists may not feel comfortable or well-trained to discuss the topic, and patients may be reluctant to bring up sexual concerns during their visits. Social media can play a role in studying sexual health and in sexuality interventions, particularly in adolescent and young adult (AYA) patients with cancer, who are more likely to seek such information online.^74^ Additionally, the ability to remain anonymous online may help patients with cancer feel more comfortable discussing sexuality.^75,76^ Social media is also used as a communication method to discuss sexuality education in LGBTQI+ communities, who are generally excluded from sexual education, indicating the potential use of social media to close the gap between the oncology community and sexuality for SGM and TGD individuals.^77^ While this remains a largely unexplored frontier, studies have utilized social media to describe the impact of cancer on sexuality.

Adams et al conducted a mixed methods study focusing on the psychosocial needs of survivors of gynecologic cancer.^78^ The authors analyzed discussion board posts made by survivors of gynecologic cancer on the American Cancer Society website and demonstrated that nearly 19% of the posts were related to the psychosocial experience of survivorship, including conversations on sexuality and intimacy.^78^ A survey conducted by Taylor et al, including, patients with multiple types of cancer, found that nearly 9 out of 10 respondents experienced changes after cancer treatment that resulted in a negative impact on their sexual health.^12^ The survey was administered electronically in a clinic setting and on social media (Facebook and Twitter) with 87% of participants expressing sexual dysfunction after cancer treatment.^12^ Breast cancer was one of the most common cancer diagnoses, and survivors reported sexual toxicities, including dyspareunia, difficulty achieving orgasm, distortion of body image resulting in intimacy issues, and infertility. Yet, only 44% of respondents had been told that cancer treatment could impact their sexuality and less than a third had been formally asked about their sexuality by a provider.^12^ The Women’s Insights in Sexual Health After Breast Cancer (WISH-BREAST) study surveyed women who had or have breast cancer on sexuality concerns and was disseminated using a social media platform (Instagram). There were 1775 respondents, highlighting that social media can serve as an effective research tool for people impacted by cancer.^32^ Research on sexuality and cancer can be enhanced through increased social media utilization, thereby leading to improved trial recruitment, and decreasing barriers to discussion in clinical settings.^79,80^ Despite this, social media currently remains a widely untapped resource. Digital health interventions using internet-based platforms, including educational information, interactive methods, and cognitive behavior therapy-based interventions, have led to improvements in sexual function, desire, and psychological well-being.^72^ However, these studies have been somewhat limited due to high drop-out rates and poor adherence rates, a common factor in digital health interventions.^72^ Proposed factors to improve engagement in the digital health space include institutional accreditation/health care practitioner endorsement, expert-led intervention development without commercial bias, security and confidentiality, platform usability, and personalization or tailoring of interventions.^72^ These factors must be leveraged in the social media space as well.

As many patients do not receive information on sexual health from their oncology providers,^16,81^ using social media for education and effective communication interventions may bridge this gap but requires healthcare professionals to be present and engaged on social media platforms that are predominantly patient facing. The WISH-BREAST study reported that 80% of respondents sought information about sexuality on social media, primarily from medical professional accounts.^32^ A growing number of medical professional accounts, patient-facing organizations, and healthcare organizations are providing this information on social media (Table 2). Drizin et al conducted semi-structured interviews to evaluate HCP perceived barriers and facilitators to social media communication about sexual health and strategies to help HCPs navigate social media use for this purpose.^82^ This was a small study of HCPs who had provided medical or supportive care to AYA cancer patients and survivors. Notable themes identified suggested that social media has the potential to facilitate patient-centered communication through a variety of ways: normalization of sexual health, encouragement to patients to engage in their sexuality care, and helping HCPs learn about the patient perspectives and needs.^82^ The importance of effective communication and learning about patient perspectives is exceedingly pertinent for SGM and TGD communities as oncology providers are often undereducated when it comes to these patient populations.^14,21^ However, challenges remain in doing this effectively such as concerns for professional social media use, lack of social media training, time constraints, and the need for brevity in social media posts.^82^

**Table 2.** Patient resources. Please note that this list is not all-inclusive, just a few suggestions.

Type	Resource
Social media	Twitter ◦ @SGMCancerCARE ◦ @cancersexnet ◦ @drteplinsky ◦ @oriordanliz ◦ @LailaAgrawalMD ◦ @NarjustFlorez Instagram ◦ @oriordanliz ◦ @drteplinsky ◦ @drlailaagrawal ◦ @kellycasperson ◦ @drmennobgyn ◦ @lgbtcancernetwork ◦ @menopause_and_cancer
Podcasts	Interlude Women’s Cancer Stories with Dr. Eleonora Teplinsky ◦ Episode 125 “Sexual Health” ◦ Episode 139 “Let’s Talk About Lung Cancer, Sexual Health, and Cancer Disparities” You Are Not Broken, Kelly Casperson Menopause and Cancer
Websites	Prosayla.com — An expert and patient reviewed source, supported by ISSWSH Scientific Network on Female Sexual Health and Cancer Cancer Support Community American Cancer Society—Sex and the Adult Female with Cancer Breastcancer.org Home (meetrosy.com) National Cancer Institute
Videos	How cancer treatment affects sexuality in women | Dana-Farber Cancer Institute — YouTube
Books	Come as You Are by Dr. Emily Nagoski Sex and Cancer Intimacy, Romance, and Love After Diagnosis and Treatment by Dr. Saketh Guntupalli and Maryann Karinch Woman Cancer Sex by Dr. Anne Katz The Better Sex Through Mindfulness Workbook by Dr. Lori Brotto You are Not Broken by Dr. Kelly Casperson

The prevalence of misinformation on social media can make it challenging for patients to identify accurate information.^83,84^ In fact, in a quality review of social media articles on cancer treatment, 32.5% of articles contained misinformation.^84^ This is a significant concern and ensuring information comes from accredited sources and is backed by providers, evidence, government organizations, and cancer centers can mitigate this.^85,86^ The use of social media by these sources can improve the amount of misinformation available as disseminating accredited information through social media improves the chances that individuals seeking information online will find it from a credited source.^84,86^ Moreover, encouraging patients to feel comfortable discussing online sexual health resources with their HCPs is one way of “vetting” the information.

## How to begin the conversation about sexuality

**Figure 2.**
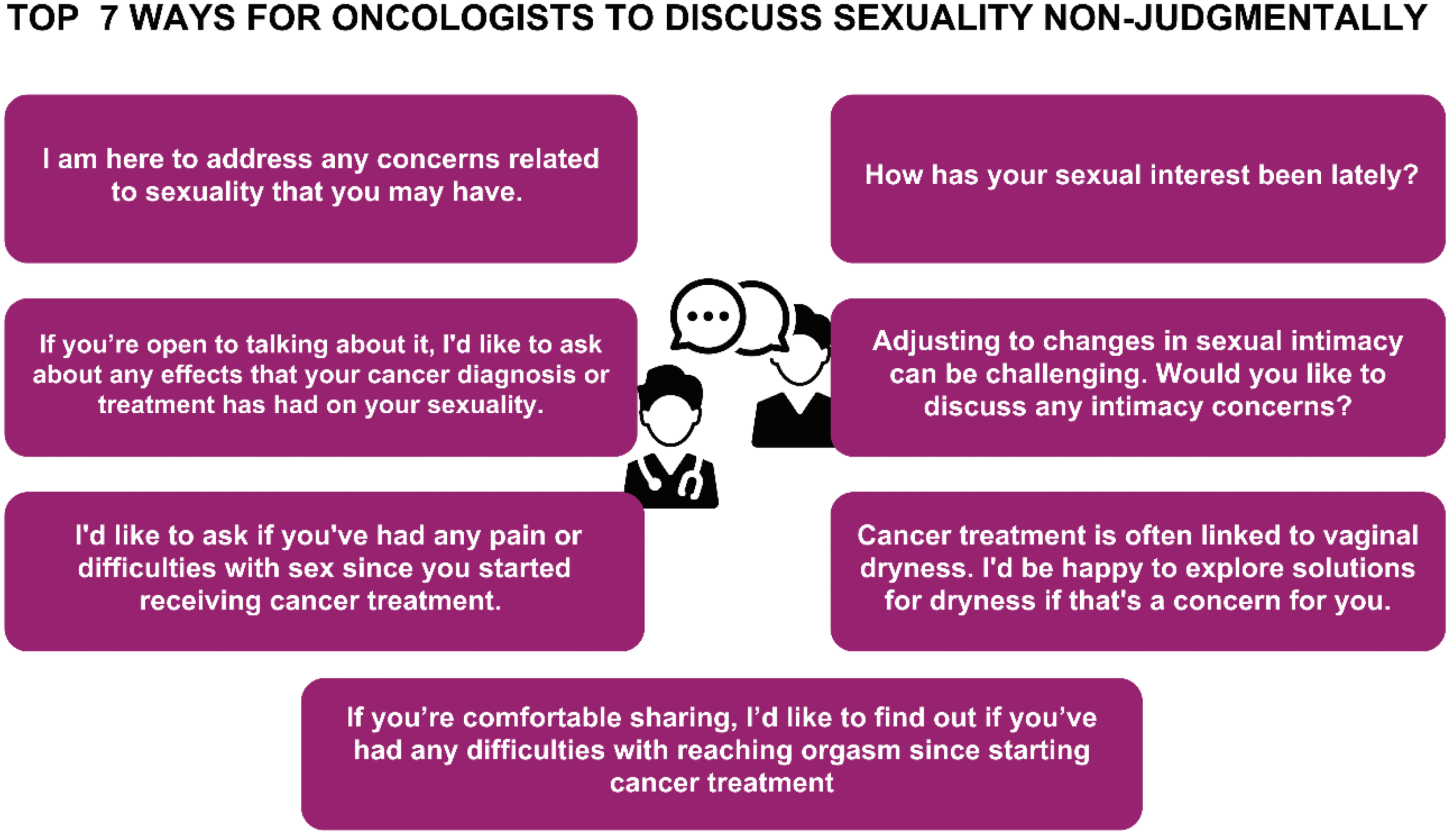
Top seven ways for oncologist to discuss sexuality non-judgmentally^36^.

**Figure 3.**
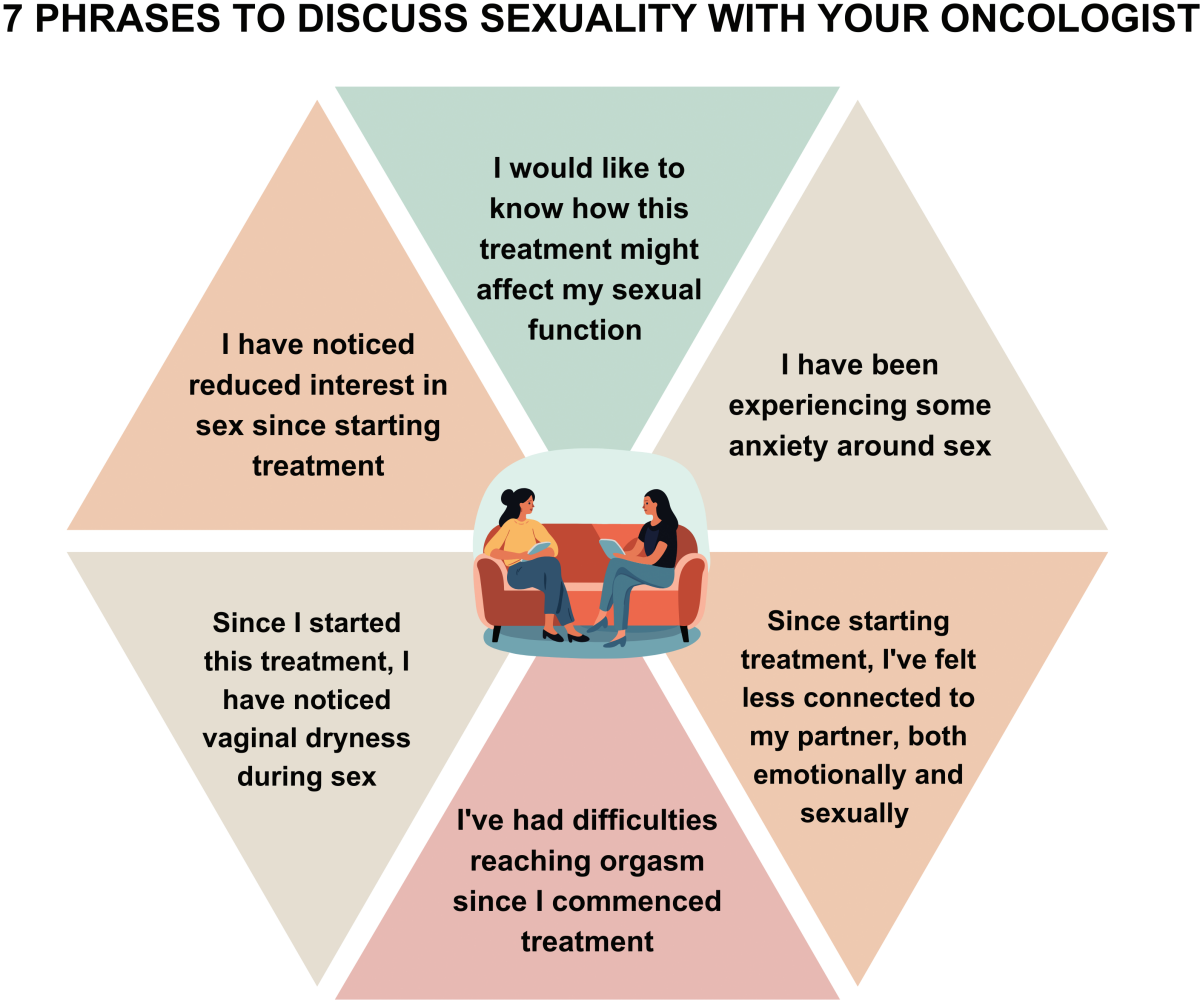
Seven phases for patients to discuss sexuality with oncologists^87^.

Providers and patients interested in starting a discussion about sexuality may need guidance on where to start the discussion.^15^ Guidelines for non-judgmental questions providers may use are available in Figure 2. Opening phrases patients may use to catalyze a conversation about sexuality are available in Figure 3. Clinicians can share this figure for patient education.

## Conclusions

Despite the existence of clinical guidelines for the treatment of sexuality concerns, this topic remains under-discussed and understudied, leading many people with cancer to be left in the dark.^2,12^ Current interventions and data on the incidence of sexual dysfunction and how it presents itself can be used to tailor programs so that they best meet the needs of varying cancer types and diverse populations. Social media is a largely untapped resource that can be used for research and to disseminate accessible information about sexuality to patients. Treatment guidelines, patient resources, and discussion frameworks as presented in this paper can provide oncologists with a framework to incorporate inclusive sexuality care in their practice.

## Data Availability

No new data were generated or analyzed in support of this research.
